# Taking balance training for older adults one step further: the rationale for and a description of a proven balance training programme

**DOI:** 10.1177/0269215514546770

**Published:** 2015-05

**Authors:** Alexandra Halvarsson, Ing-Mari Dohrn, Agneta Ståhle

**Affiliations:** 1 Karolinska Institutet, Department of Neurobiology, Care Sciences and Society, Division of Physiotherapy, Sweden; 2 Department of Physical Therapy, Karolinska University Hospital, Sweden

**Keywords:** Balance, exercise, older adults, training programme

## Abstract

**Objective:**

To give the rationale and evidence for and a detailed description of a rehabilitation programme of proven effectiveness in improving balance in older adults.

**Background theory and evidence:**

Based on the knowledge that balance loss usually occurs in situations when attention is divided, especially when being older, and that balance control relies on the interaction of several physiological systems, we have developed a specific and progressive balance training programme with dual- and multi-task exercises for older adults.

**Practical application:**

Balance demanding exercises, specific to the various components of balance control and to situations in daily life, were performed in sitting, standing and walking at three different levels of progression (basic, moderate and advanced) of increasing difficulty and complexity. The training was performed in 45-minutes group sessions, with 6–10 participants in each group, three times per week during 12 weeks, with two or three physiotherapists present.

**Conclusions:**

This balance training programme strengthens self-efficacy in balance control leading to improved fall-related self-efficacy, reduced fear of falling, increased walking speed, and improved physical function. Participants found the programme motivating, valuable, fun, and enjoyable, which was reflected in a high attendance rate.

## Introduction

We have developed a balance training programme for older adults aiming to reduce fear of falling and to improve their balance function in daily life, thereby contributing to better health. In this article we give the rationale for this programme by reviewing the theory and evidence underlying this programme, and give details on its implementation in real practice.

Balance control is the foundation of a person’s ability to move and function independently. However, balance control declines with age, and impaired balance is a major risk factor for falls among older adults.^1^ A fall can result in severe injuries, such as fractures, causing longstanding pain, lower quality of life, disability, and even death.^2^ Fear of falling, with or without an actual previous fall, can lead to physical inactivity^3^ accompanied by further physical decline and impaired balance, as well as to an increased risk of many lifestyle-related diseases.^4,5^ Moreover, previous research has found that fear of falling per se may increase the risk of a future fall.^6^ Dual-task performance, or performance when a person’s attention is divided between a motor and a cognitive task, is a natural component of daily activities. Both healthy elderly persons and those with balance impairments show reduced physical performance under cognitive stress or while engaging in attention-demanding tasks, increasing the risk of falling even more.^1^

Training balance during dual-task conditions appears to be necessary to improve balance control under situations with divided attention, as balance training with single-task exercises have shown to not transfer to dual-task performance.^7^ To date, as reported in the literature, there is only weak evidence that some types of exercises, such as gait, balance, co-ordination, and functional tasks, are moderately effective in improving balance in older adults and that further methodologically high-quality research is required.^8,9^

This balance training programme for older adults, including dual- and multi-task exercises, was designed and developed based on well-established principles of exercise and on the knowledge that balance control relies on the interaction of several physiological systems, as well as interaction with environmental factors and the performed task.^10–12^

Two randomized controlled studies have found this balance training programme to be motivating and valuable in strengthening self-efficacy in balance control, resulting in a reduced fear of falling, increased walking speed, and improved physical function.^13–15^ Participants also found the programme fun and enjoyable, which was reflected in a high attendance rate.

## Evidence and theory

The ability to stand, to walk and to perform daily activities in a safe manner depends on a complex interaction of physiological mechanisms and many systems have influence on a person’s balance.^16^ A model summarizing six systems responsible for poor functional balance has been published to help therapists target specific types of interventions for different types of balance problems.^17^ Our programme includes exercises aiming to stress and improve five of this model’s six systems or domains (excluding biomechanical constraints) i.e. stability limits, anticipatory postural adjustments (APA), postural responses, sensory orientation (somatosensory, visual, and vestibular), and stability in gait (dynamic balance), see supplementary material Figure 1.

Balance control also requires many cognitive resources, and the more difficult the postural task, the more cognitive processing is required.^18^ Balance control and other cognitive processing share cognitive resources and, therefore, simultaneous performance of a secondary task affects stability in both healthy and balance-impaired older adults. Performance levels vary depending on the complexity and type of the secondary task.^19,20^ Furthermore, a secondary task affects balance, gait, and fall risk,^21^ especially in older adults. Since dual-tasking has a major impact on daily physical performance, we believe that dual- and multi-task exercises are important components in a balance training programme.

According to basic exercise physiology principles, training adaptions are specific for the system trained^10,11^ and training specificity is also a key element of motor learning.^22^ In addition, evidence supports the effectiveness of specific training to improve postural control among healthy older adults during dual-task conditions.^7^ This programme follows the principle of specificity in that it is based on exercises targeting various systems for balance control (see supplementary material Figure 1). It also aims to improve balance performance in specific situations that can occur in daily life, such as regaining postural stability after perturbation or being able to suddenly avoid an obstacle, with retained balance, while simultaneously walking and answering a question.

Our programme is progressive as the exercises can be performed at different levels (basic, moderate, and advanced), making it progressively challenging for each individual throughout the whole programme. This progression follows both the taxonomy of tasks,^23^ which explains how each individual can improve his or her skills by performing training tasks of increasing complexity, and the basic concept of progression in exercise physiology, meaning that the intensity, difficulty, or complexity of exercises need to be increased as the body adapts to exercise over time.^10,11^

Even though the exercises in the programme primarily target the domains body functions and activities, according to the International Classification of Functioning, Disability and Health (ICF),^24^ we believe that the programme can also influence the domain participation through increased activity in daily life.

## Practical application

We chose to perform the exercises as a group programme, in 45-minutes group sessions, three times a week during 12 weeks as this has proved to be valuable from several points of view. A systematic Cochrane review, found that the most effective balance training programmes were those running three times a week for a duration of three months, involving standing, challenging balance exercises.^9^ The review did not compare group training programmes versus individual training, however, comparison between dual-task exercise interventions have demonstrated similar improvements both in group and individual training.^7^ Furthermore, exercise in groups provide a social belonging, support and identification with people having the same problem (which may contribute to an increased attendance rate) and also shown to be cost-effective.^25,26^ To optimize the training outcome and social interaction we chose to have groups of 6-10 participants in each group. To ensure the participants’ safety and to make it possible to adjust and individually progress the exercises to the point of balance loss, avoiding external support from the physiotherapist to allow the natural balance response to execute fully, two or three physiotherapists were present at each session. Based on our experience, we recommend that a group consist of eight participants with two physiotherapists present.

Each session started with a short (5-7 minute) warm-up (marching in place, one-leg standing, weight shifting, lunges, arm and neck movements), followed by exercises performed while sitting on balls (15 minutes) and exercises standing and walking (15-20 minutes). The sessions ended with short stretching and breathing exercises. The sitting, standing, and walking exercises differed among sessions to obtain variety, but every exercise returned later in the programme, often in a more difficult form. See Tables 1 to 4 for examples of exercises and added motor and cognitive tasks (dual- and multi-task activities). During exercises with and without dual- and multi-tasking, the participants were able to reflect on the difference in their performance.

**Table 1.** Exercises sitting, standing, and walking at basic, moderate, and advanced levels in the balance training programme.

	Basic	Moderate	Advanced
Exercises sitting on a ball	• Sitting • Sitting in a circle and passing (rolling) a large ball • Sitting in a circle, kicking a ball (supplementary material Figure 2) • Sitting in a line, passing a ball • Sitting in a line, rolling a large ball, slalom • Sitting in pairs facing each other, hands together, pushing gently*	• Sitting, adding a motor or cognitive task • Sitting in a circle and passing (rolling) a large ball, one foot on cushion • Sitting in a circle, kicking a ball, feet or arms in different positions (supplementary material Figure 2) • Sitting in a line and passing a ball, one foot on cushion • Sitting in a line, rolling a large ball, slalom, one foot on cushion • Sitting, parrying a gentle push*	• Sitting, adding both a motor and cognitive task • Sitting in a circle and passing (rolling) a large ball, both feet on a cushion • Sitting in a line and passing a ball, both feet on a cushion • Sitting in a line, rolling a large ball, slalom, both feet on a cushion

* Exercises excluded for participants with osteoporosis.

**Table 2.** Exercises standing at basic, moderate, and advanced levels in the balance training programme.

	Basic	Moderate	Advanced
Exercises standing	• Standing in a circle, chairs in between, passing (rolling) a large ball • Standing in a circle, passing around a big balloon • Standing in a line, passing a small ball, slalom • Standing in a line, passing (rolling) a large ball, slalom (supplementary material Figure 3) • Tandem stance • Standing on one leg • Standing, one balance cushion under each foot • Standing, both feet on one balance cushion • Standing on soft foam mat • Standing in pairs, facing each other, both holding a ball. One leads and the other follows when moving the ball in the air • Standing in pairs, facing each other with a ball between chests, pushing gently*	• Standing in a circle, hitting a balloon, doing a lunge when hitting • Standing in a circle, passing a ball, doing a lunge when passing ball • Standing in a circle, throwing a ball, doing a lunge when throwing (one or two balls • Standing in a circle, throwing a ball with different bases of support (feet together, tandem stance, etc.) • Standing in a circle, passing around a glass of water, one foot on balance cushion • Standing in a line, passing a small ball, slalom, with different bases of support (one foot on balance cushion, semi-tandem, etc.) • Standing in a line, passing (rolling) a large ball, slalom, with different bases of support (one foot on balance cushion, semi-tandem, etc., supplementary material Figure 3) • Standing with one hand lightly resting on back of chair for support, one foot on balance cushion, doing lunges with other leg in different directions • Standing with different bases of support (tandem stance, standing one balance cushion under each foot, both feet on one balance cushion), adding motor or cognitive task	• Adding a cognitive task (reciting or counting) to the exercises in moderate level • Standing with different bases of support (tandem stance, standing one balance cushion under each foot, both feet on one balance cushion), adding both motor and cognitive tasks

* Exercises excluded for participants with osteoporosis.

**Table 3.** Exercises walking at basic, moderate, and advanced levels in the balance training programme.

	Basic	Moderate	Advanced
Exercises walking	• Slalom walking around 4–7 balance cushions • Walking, stepping with one foot on balance cushions (4–7 cushions) • Walking around in a “messy” surrounding (chairs, balls, steps, cones, etc.) • Walking forward at a fast speed and returning walking backwards • Walking forward at a fast speed, finishing with stepping up and down on two step platforms, returning at normal speed • Walking forward in fast speed, finishing with stepping up and down on two step platforms, returning walking backwards • Walking on a long soft foam mat • Semi-tandem or tandem walking	• Slalom walking, adding motor or cognitive task (supplementary material Figure 4) • Walking, stepping on balance cushions, placed in a row wide apart • Walking around in a “messy” surrounding (chairs, balls, steps, cones, etc.), reciting or counting • Walking around, doing lunges on request (with left foot when tapped on left shoulder) • Walking forward at a fast speed and returning walking backwards, adding motor or cognitive task • Walking forward at a fast speed, finishing with stepping up and down on two step platforms, returning at a normal speed, adding motor or cognitive task (supplementary material Figure 6) • Walking forward at a fast speed, finishing with stepping up and down on two step platforms, returning walking backwards, adding motor or cognitive task • Walking on a soft foam mat, adding motor or cognitive task • Tandem walking, adding motor or cognitive task	• Slalom walking, adding both motor and cognitive task • Walking, stepping on balance cushions placed in a row (supplementary material Figure 5) • Walking around buttoning and unbuttoning clothing or reciting or counting in a “messy” surrounding, doing lunges on request (with left foot when tapped on left shoulder) • Walking forward at a fast speed and returning walking backwards, adding both motor and cognitive task • Walking forward at a fast speed, finishing with stepping up and down on two step platforms, returning at a normal speed, adding both motor and cognitive task • Walking forward at a fast speed, finishing with stepping up and down on two step platforms, returning walking backwards, adding both motor and cognitive task • Walking on a soft foam mat, adding both motor and cognitive task • Tandem walking, adding both motor and cognitive task

**Table 4.** Examples of added motor and cognitive tasks (dual- and multi-task activities) included in the training programme.

***Motor task***
Moving arm, leg, head, or trunk (leaning, turning)
Buttoning and unbuttoning clothing
Juggling a balloon
Throwing and catching a ball
Kicking a ball
Carrying a glass of water, a tray with several glasses of water, or a large ball
Rolling a Ping-Pong ball on a tray
Closing eyes
***Cognitive tasks***
Counting - adding or subtracting by three or seven from a given start number
Reading a newspaper, silently or aloud
Reciting categories of flowers, animals, countries, cities, names, etc.

The balance training was performed in an exercise room at a physiotherapy department. We recommend a room approximately 50 square meters.

The following supplies were used for this training programme:

- Different-size balance balls (55-75 cm diameter) used during exercises in sitting.
- Balance-disc cushions (34 cm diameter) and soft foam mats (5 cm thick and 4 m long)  to make surface uneven.
- Two height adjustable step platforms.
- Soft foam balls (20 cm diameter) used for throwing, catching, and kicking.
- Balloons.
- Armchairs to place between subjects to ensure safety and balance support if necessary.
- Trays.
- Drinking glasses with water.
- Newspapers.

The following list outlines the progression of the balance training:

- Basic level: One to two components are in-corporated.
- Moderate level: Three to four components are incorporated.
- Advanced level: More than four components are incorporated, almost always including  both a motor task and a cognitive task (dual- or multi-task activities).

For example:

At the basic level, participants walk on uneven surfaces (dynamic balance and sensory orientation), at the moderate level they walk a course with reduced step width (stability limits, dynamic balance, and sensory orientation), and at the advanced level a cognitive dual-task is added (stability limits, dynamic balance, sensory orientation, dual- and multi-task activities and postural responses).

Basic level: Sitting on a ball with a large base of support.

Moderate level: Sitting on a ball and adding a motor task, such as juggling a balloon (dual-task), with a reduced base of support or an uneven surface under the feet.

Advanced level: Sitting on a ball with a reduced base of support and adding a motor task or a cognitive dual-task, or combining a motor task and a cognitive task (buttoning and counting/head movements/eye closed). Sitting on a ball with a reduced base of support and juggling a balloon, and adding an uneven surface under the feet.

Basic level: Walking on even ground while performing a motor task or a cognitive task.

Moderate level: Walking with reduced-step width while performing a motor task or a cognitive task, or walking and combining a motor task and a cognitive task (carrying a tray while talking), or performing two motor tasks (carrying a tray while performing head movements).

Advanced level: Walking with reduced-step width and combining a motor task and a cognitive task or performing two motor tasks.

Even though the balance training was performed in group, the exercises were individually adjusted for each participant with the aim of constantly challenging the balance control systems. These examples demonstrate how the exercises can be individually adjusted and challenging for each individual:

- Using different arm and foot positions: Arms hanging at the sides or crossed over the chest or placed in the lap while sitting. For changes in support area, the feet can be placed wide apart, near each other, close together, in a semi-tandem or tandem stance, or on balance-disc cushions (see supplementary material Figure 7).
- Performing exercises at different speeds, such as walking, head movements, or arm and leg movements.
- Varying the spacing between the cushions or varying the number of cushions when walking on balance-disc cushions.
- Adding a motor task or/and a cognitive task. Adjusting the added task’s level of difficulty.
- Varying the density and size of uneven surfaces.

Supplementary material Figure 8 illustrates ways that an exercise can be individually adjusted.

## Discussion

The effectiveness of this programme has been evaluated in two randomized controlled studies including community-dwelling older adults with or without diagnosed osteoporosis^13–15^ showing that this balance training is successful in reducing participants’ fear of falling. However, in the future, the programme could be developed to include components that specifically address this concern. For example, therapists could discuss the fear of falling during the training sessions, allowing participants to explain their perceived fear of falling, and situations in which the participants perceive a fear of falling could be incorporated into the training programme.

The distribution of the exercises included in the programme could also be taken into consideration in the future. Exercises focused on standing and walking could be prioritized over sitting ones to make the programme more specific to balance performance during walking and standing, thereby better reflecting balance tasks in daily life.

This balance training programme can easily be modified to suit different conditions - for example, by excluding exercises with pushes and trunk rotations for older adults with osteoporosis, as noted in Table 1. To optimize the training’s effectiveness, it is important to customize the balance training for each individual and to make personalized adjustments during the group sessions, as well as to consider personal factors and coping strategies.

The balance training programme may be used either in clinical practice exactly as presented or as a guidance structure for group balance training programmes. It can be modified to suit available resources, local conditions (such as type of equipment and localities), and other patient groups. If the training programme is modified, it should remain grounded in the theoretical background of balance control, basic exercise principles, and increasing difficulty - that is, it should always incorporate progressively more complex exercises involving both motor and cognitive tasks (dual- and multi-task activities), as described.

To ensure that the balance training is effective, we believe that two or more physiotherapists, depending on the number of participants and their balance abilities, should be present to facilitate the individual progression of exercises and to challenge each participant in a safe way.

**Clinical messages**

- This balance programme improves balance control, fall-related self-efficacy, fear of falling, walking speed, physical function and can easily be modified to suit different conditions.
- If the programme is modified, it should remain grounded in the theoretical background of balance control, basic exercise principles, and involving both motor and cognitive tasks.

## Supplementary Material

Supplementary material
