# Supplementary material for: Taking balance training for older adults one step further: the rationale for and a description of a proven balance training programme
Source: Clin Rehabil. 2015 May;29(5):417–25. doi: 10.1177/0269215514546770 (PMC4419050; doi:10.1177/0269215514546770)
Supplement: Supplementary material [file Online_Figures.pdf]

Figures:

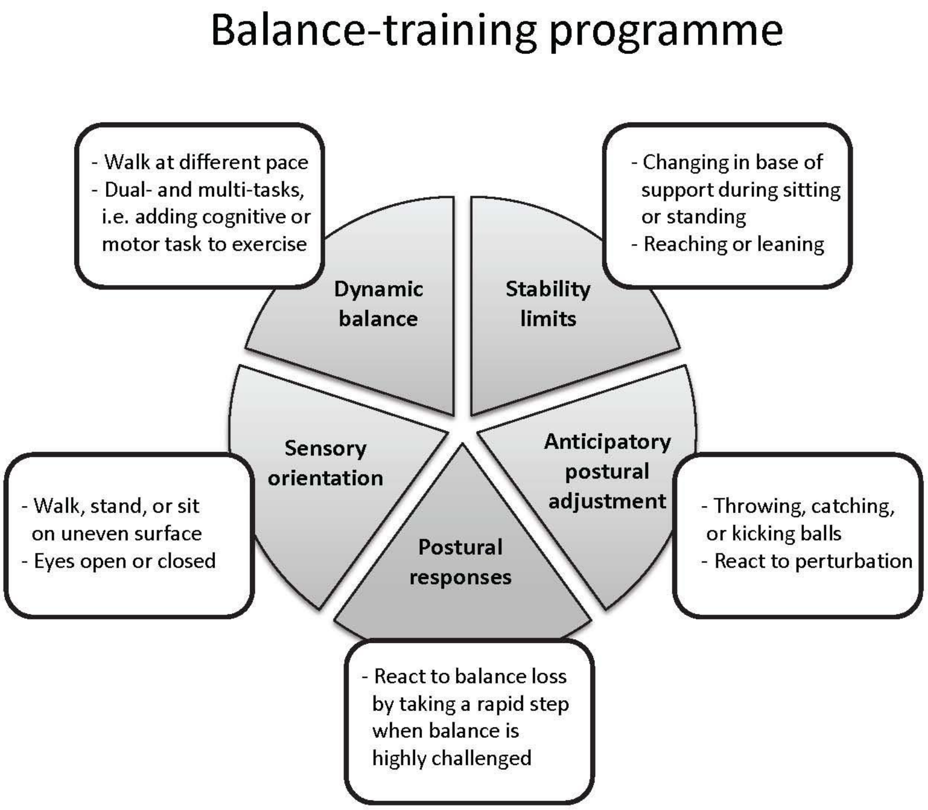

Figure 1. Illustration of the balance training programme and the various components in the programme associated with the different systems that contribute to balance control.

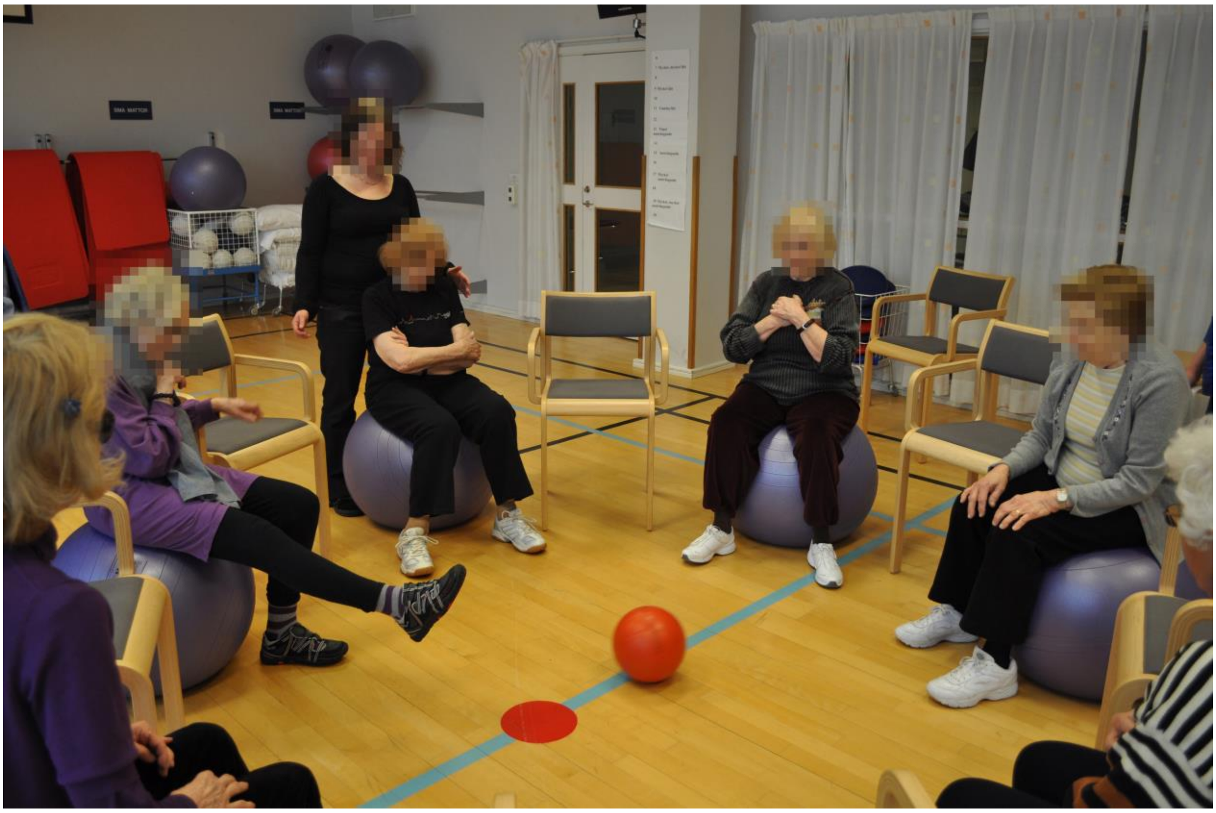

Figure 2. Sitting in a circle, kicking a ball, holding arms in different positions (crossed over the chest or placed in the lap).

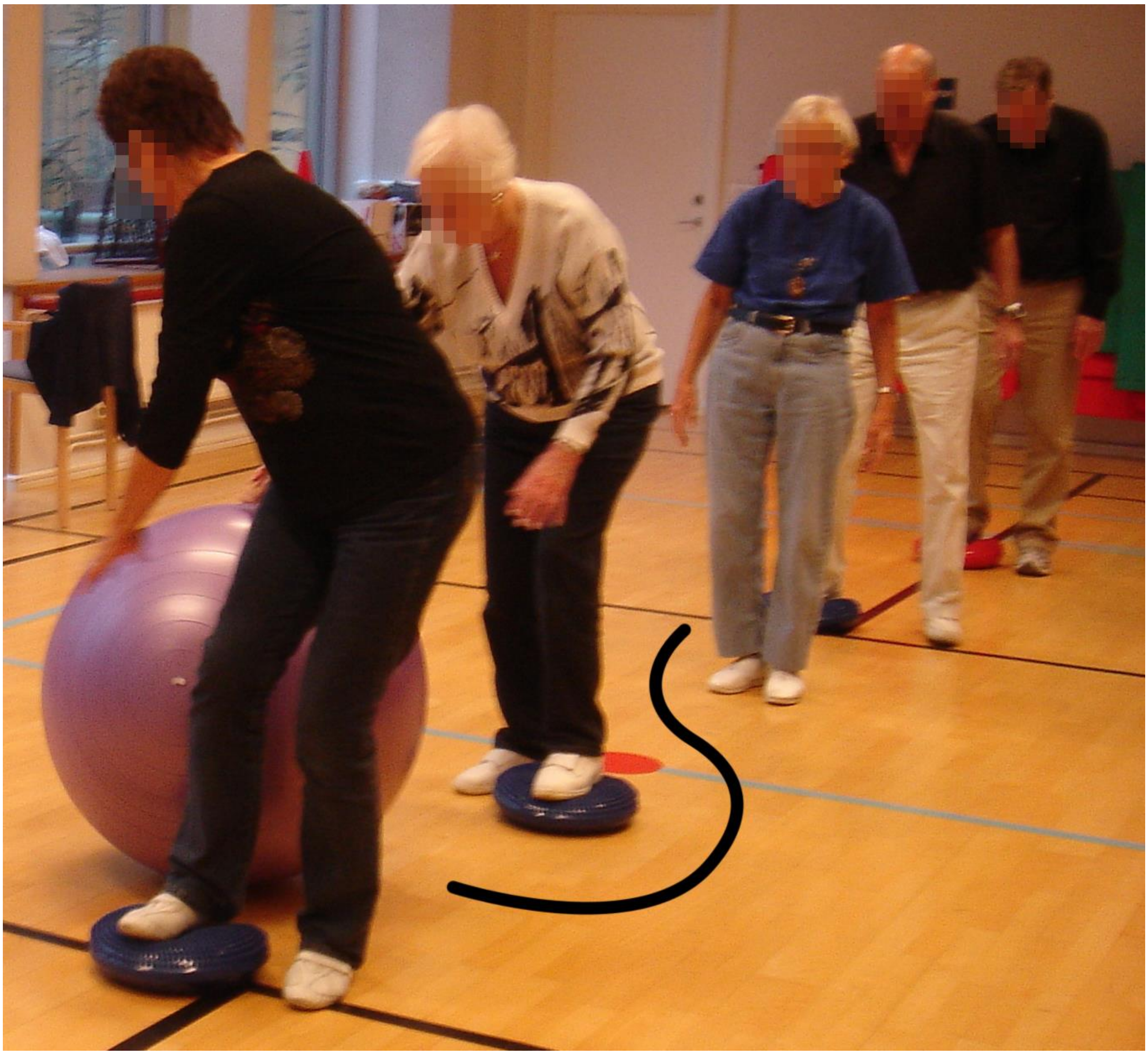

Figure 3. Standing in a line, passing (rolling) a large ball, slalom, with different bases of support (feet close together or one foot on balance cushion).

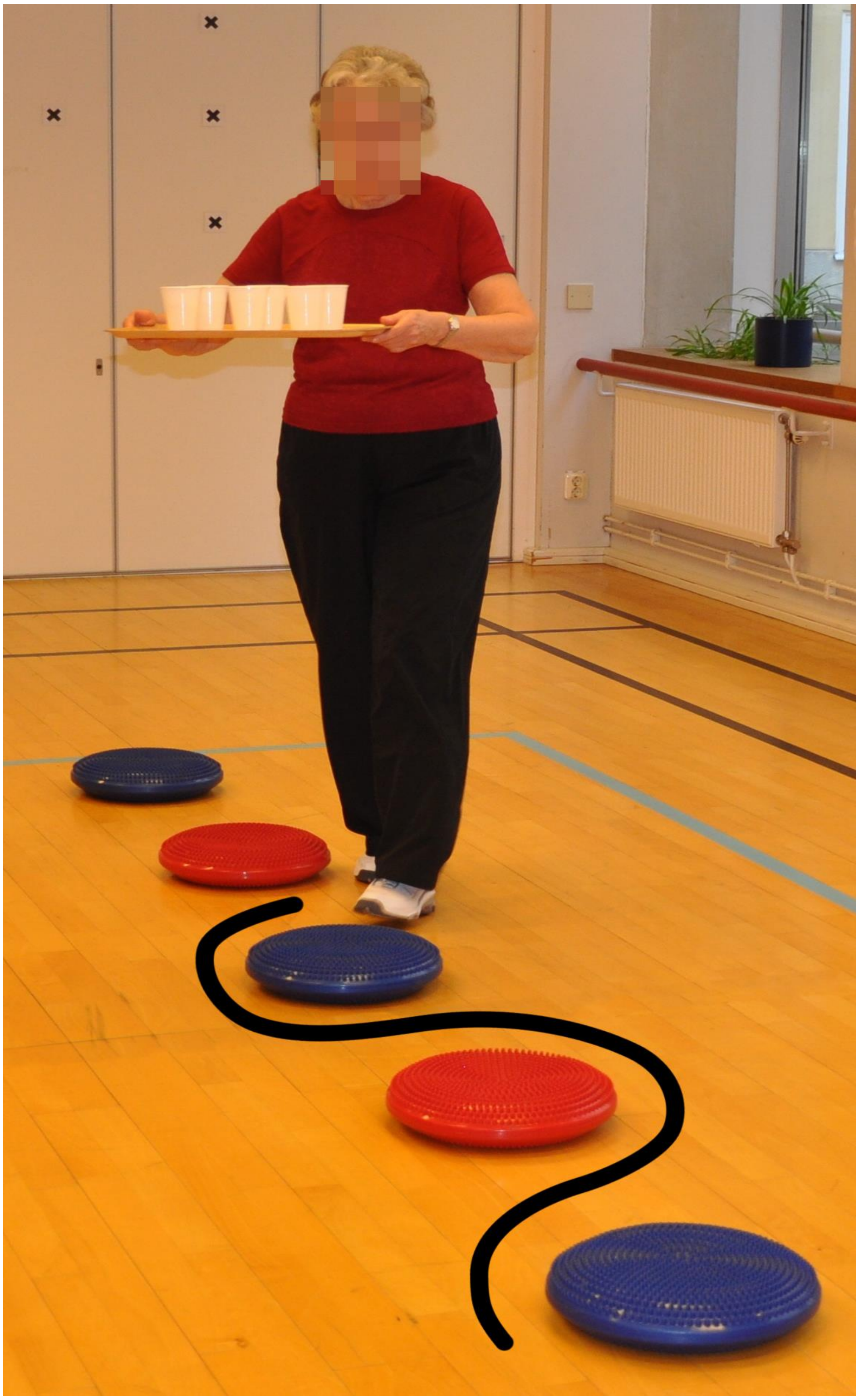

Figure 4. Slalom walking around five balance-disc cushions, placed in a row wide apart, while performing an added motor task (carrying a tray of drinking glasses).

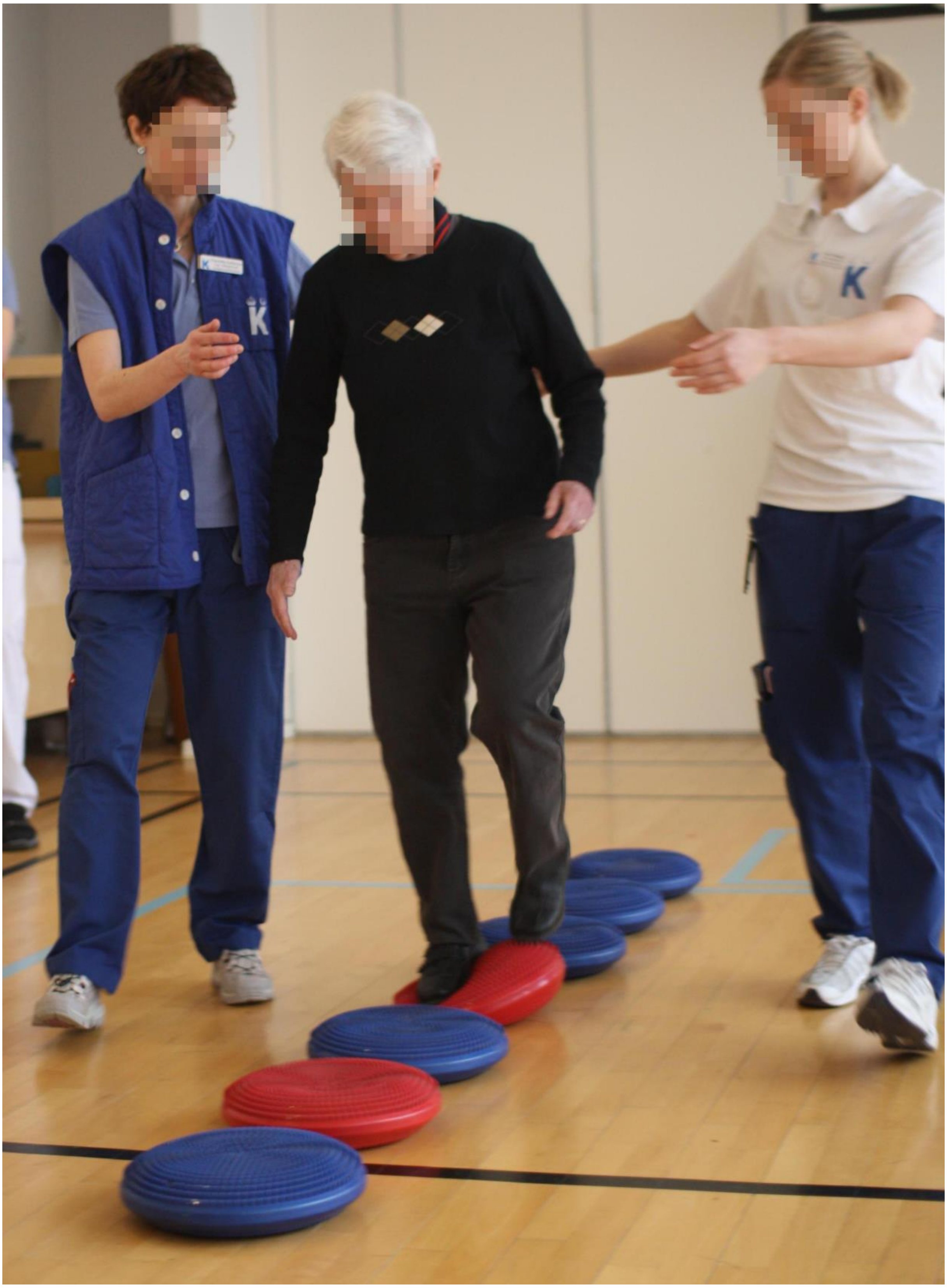

Figure 5. Walking, stepping on four to seven balance cushions placed in a straight line, touching or apart. Step with one foot on the cushion or with both feet.

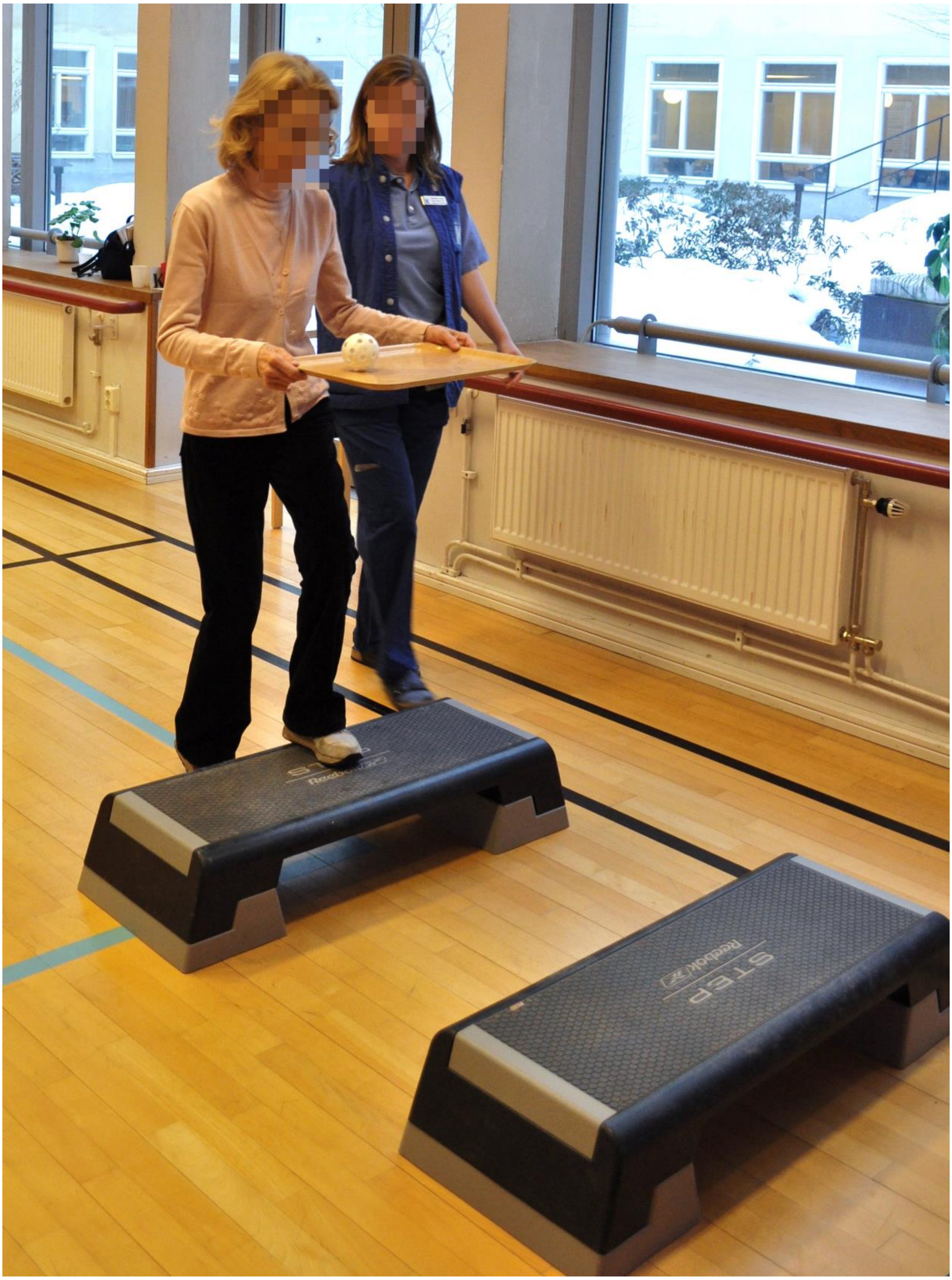

Figure 6. Walking forward at a fast speed, finishing with stepping up and down on two height-adjustable step platforms while performing a motor task (rolling a Ping-Pong ball on a tray).

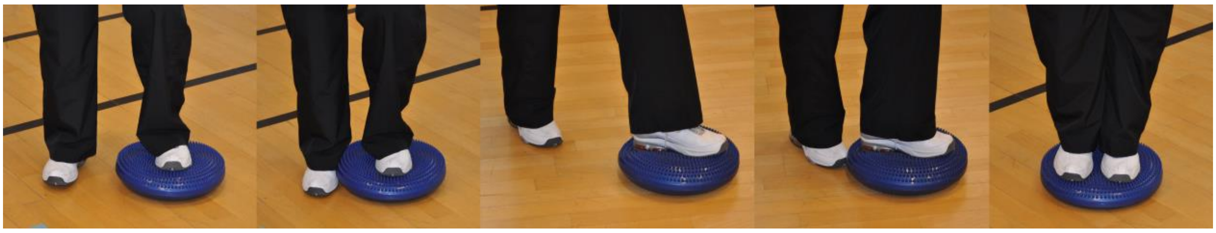

Figure 7. Different foot positions using a balance-disc cushion.

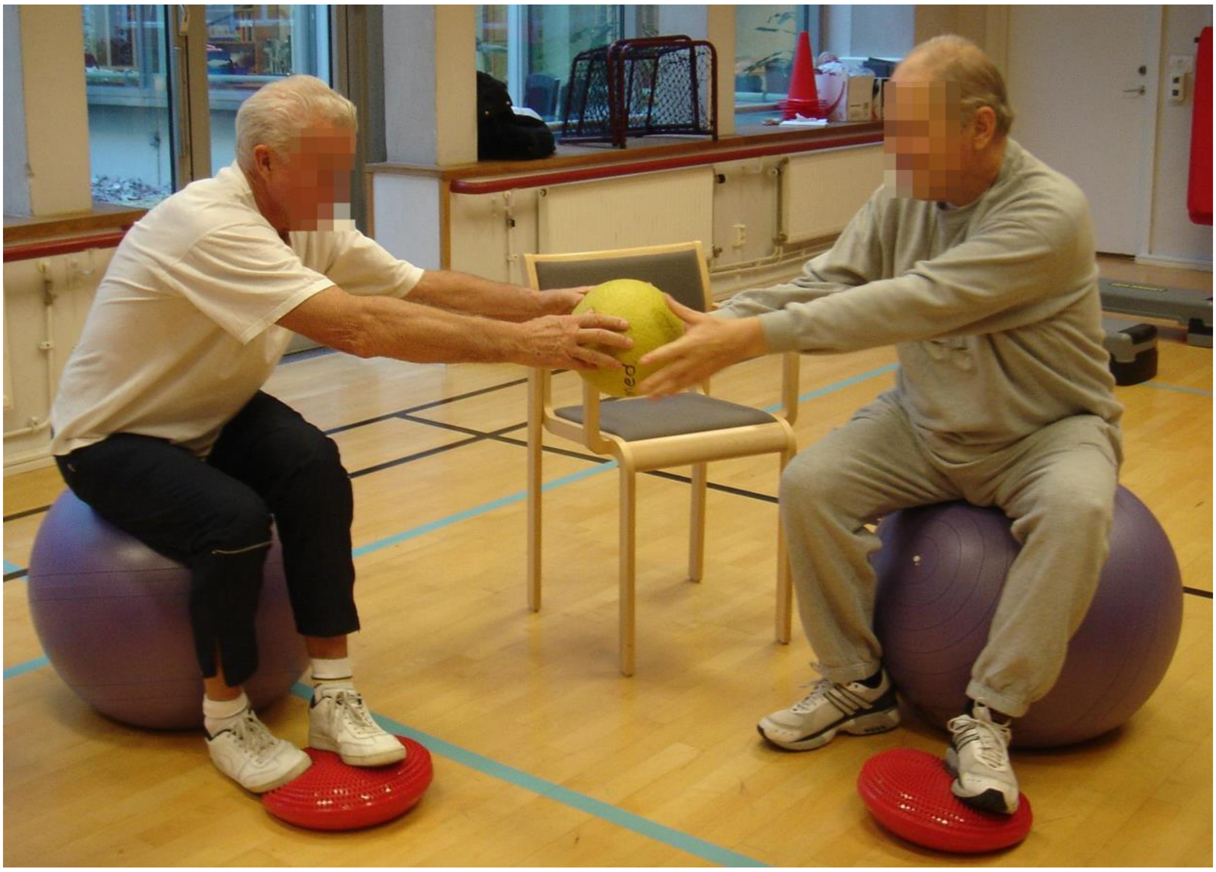

Figure 8. Two participants performing a balance-demanding exercise: sitting on balls and passing round a small ball. The participant to the left has both his feet on an uneven surface with a small base of support and is leaning towards the other participant. The participant on the right has one foot on an uneven surface, has a wide base of support, and is sitting more upright.
